# Reducing Time to First on Scene: An Ambulance-Community First Responder Scheme

**DOI:** 10.1155/2016/1915895

**Published:** 2016-03-31

**Authors:** Alan Campbell, Matt Ellington

**Affiliations:** ^1^School of Clinical Medicine, Addenbrooke's Hospital, University of Cambridge, Hills Road, Cambridge CB2 0SP, UK; ^2^Maidstone and Tunbridge Wells NHS Trust, Tonbridge Road, Tunbridge Wells, Kent TN2 4QJ, UK

## Abstract

The importance of early access to prehospital care has been demonstrated in many medical emergencies. This work aims to describe the potential time benefit of implementing a student Community First Responder scheme to support ambulance services in an inner-city setting in the United Kingdom. Twenty final and penultimate year medical students in the UK were trained in the “First Person on Scene” Business and Technology Education Council (BTEC) qualification. Over 12 months, they attended 89 emergency calls in an inner-city setting as Community First Responders (CFRs), alongside the West Midlands Ambulance Service, UK. At the end of this period, a qualitative survey investigated the perceived educational value of the scheme. The mean CFR response time across all calls was an average of 3 minutes and 8 seconds less than ambulance crew response times. The largest difference was to calls relating to falls (12 min). The difference varied throughout the day, peaking between 16:00 and 18:00. All questionnaire respondents stated that they felt more prepared in assessing and treating acutely unwell patients. In this paper, the authors present a symbiotic solution which has both reduced time to first on scene and provided training and experience in medical emergencies for senior medical students.

## 1. Introduction

Early access to medical care is an important factor in ensuring positive patient outcomes in emergency situations. This has been reinforced by the* Chain of Survival* concept in out-of-hospital cardiac arrests [1], in which significant improvements in survival rates have been achieved by early access to medical care and defibrillation, which reflects the importance of minimizing the delay to first on scene medical intervention [2]. The importance of prehospital care in traumatic brain injury has also been demonstrated, in which it may lead to improved postresuscitation neurological status [3]. However, in the United Kingdom (UK), recent changes in the access and supply of emergency services have led to increased caseloads for ambulance crews [4]. Although some UK services have seen additional funding for extra front-line staff [5, 6], further, more flexible approaches may be beneficial.

UK ambulances respond to emergency calls, major incidents, and urgent admission requests from clinicians and provide high dependency and urgent interhospital transfers. Emergency calls are handled by dispatch teams who ascertain the urgency of the calls and allocate ambulance resources as appropriate.

Community First Responder (CFR) schemes aim to provide rapid response to emergency calls. CFRs are trained to the level of First Person on Scene First Aid, including oxygen therapy and airway adjuncts and manoeuvres. By attending scenes of emergency prior to the arrival of an ambulance, CFRs may help improve patient outcomes, including improving survival rates in cardiac arrests by 10% per minute of CFR attendance ahead of ambulance arrival [7]. CFRs may provide valuable support to ambulance services; however, quantitative evidence in support of CFR schemes reducing time to treatment is relatively lacking.

Prehospital care delivered by trained persons is an essential aspect of modern emergency medicine. Despite this, there is a relative paucity of undergraduate-level training for medical students in this area, with only a small number of specialized programs in the UK [8, 9]. Providing prehospital care training and experience, through a first responder program, both supplement medical student learning in emergency medicine in addition to supporting ambulance services. Before such a service can be implemented, an evidence base of student-supported prehospital care must be generated.

The aim of this retrospective case analysis was to determine whether any significant reduction in time to first-on-scene treatment could be achieved by the use of a medical student CFR scheme to a county ambulance service. Additionally, the educational value of the scheme, as perceived by the students, was investigated.

## 2. Methods

### 2.1. Study Design

The study was a service evaluation carried out as a retrospective case analysis of CFR dispatch and response times and ambulance response times.

### 2.2. Study Setting

Final and penultimate year medical students with an interest in prehospital care or acute medicine from the University of Birmingham, UK, were trained in extended first aid to the level of the preexisting “First Person on Scene” Business and Technology Education Council (BTEC) qualification by the West Midlands Ambulance Service (WMAS). Training was in the form of a full-time, 16-hour, accelerated program that provided CFRs with the skills required to provide basic on-scene emergency care. This training also provided 2 observer shifts with the ambulance service. Students interested in the scheme filled in an application detailing their commitment and experience to prehospital and acute care. Although there was no requirement for prior prehospital care or ambulance training or experience as this is not a standard part of the student curriculum, some students did possess this through avenues such as medical electives.

This scheme had run for two years previously. This study was a service evaluation of this scheme, retrospectively evaluating the quality of care provided in terms of response times of CFRs in comparison to those of the ambulance service.

All students attended a briefing discussing the potential risks and benefits to relevant stakeholders, given by the scheme's organizers and ambulance service staff, prior to formal application to the scheme. CFRs were able to leave the scheme at any point during training or active service without penalty, with 2 (of 20, representing 90% retention) choosing to do so. There was no obligation on the CFRs to provide continuous 24/7 service; rather, the scheme was provided on a best effort basis. Data was collected and anonymized for the analysis as part of usual service evaluation, with CFRs providing implied consent through application following the briefing at which this was discussed.

In line with standard UK undergraduate medical training, all students had previously attained the “Basic Life Support” qualification. Additionally, students had attended the “Undergraduate Prehospital Trauma Course,” a prehospital care course provided by the South Birmingham Trauma Unit (Selly Oak Hospital, Birmingham, UK) and accredited by the Faculty of Pre-Hospital Care (The Royal Colleges of Surgeons of Edinburgh, UK).

The students acted as CFRs, working alongside WMAS, in inner-city Birmingham, UK, a well-developed urban environment with good transport links. The CFR group attended local emergency services calls requesting ambulance attendance, with the aim of reducing the time delay between the placing of the call and the arrival of assistance.

CFRs were issued with ambulance service issued identification and high visibility clothing. A generic “First Responder Kit Bag” was provided, which contained equipment and consumables up to the scope of capabilities of a CFR: basic medicines, such as oxygen; life support equipment such as an automatic external defibrillator; observations equipment such as a sphygmomanometer; basic wound care items such as dressings. These were kept stocked by the CFRs. Ambulances were equipped with GPS navigation systems; however, CFRs were not. The CFRs attended calls using their own modes of transport—such as bicycles and cars—providing the mode of transport was safe in the road conditions as per the CFRs' judgement. CFRs did not have direct communication with ambulances; rather, they had telephone contact with ambulance dispatch and control.

### 2.3. Study Protocol

Of all calls received by WMAS, CFRs responded to those meeting all of the following criteria:

- The CFR was geographically closer to the call location than the nearest ambulance.
- The CFR was within a 5-mile radius of the call location.
- The call requested medical assistance.
- The call location was not in a public area.
- The call was not to a setting posing a significant risk to CFR safety, such as calls resulting in violence.
- CFRs were able to contact the dispatching office and upgrade the call to a higher urgency if clinically indicated.
- CFRs were able to decline calls.

The limitations to calls attended were decided upon by the ambulance service as a means to mitigate risk to both CFRs and patients. Risk was also mitigated by requiring all CFRs to work within their limits of competence and the nationally agreed scope of practice (as defined by the qualifications discussed above) and requiring ambulances to be dispatched regardless of CFR allocation.

### 2.4. Study Population and Sample Size

Over a 12-month period, 89 calls under the CFR call signs were received within the Birmingham (UK) inner-city area. All of these calls were attended by the CFRs. As ambulances were dispatched as per the standard procedure, this scheme provided no additional risk or compromise to patient safety. A retrospective case analysis was performed on the data collected from each of these calls. The following data parameters were recorded:

 Call sign.
 Incident date.
 Chief complaint.
 CFR allocation time.
 CFR arrival time.
 Ambulance arrival time.

### 2.5. Study Measurements and Analysis

Every call received was logged, to enable service evaluation, and included in the analysis. Response times for ambulances and CFRs were calculated as the time from CFR allocation to arrival on scene. Response time differences between CFR and ambulance were calculated from the CFR allocation time to the respective arrival times. Data was obtained from the West Midlands Ambulance Service Information Governance Office and analyzed using statistical methods, including the *t*-test, on Microsoft Excel 2010 (Microsoft Corporation, Redmond, Washington, USA).

At the end of the 12-month period, a qualitative survey was distributed to all student CFRs and completed by 12, representing a return rate of 60%. This survey was distributed electronically, with electronic written explanation of its purpose, scope, and confirmation of consent. The survey queried the perceived educational value of the formalized training and CFR experience.

Information relating to the condition of the patient and care provided was logged; however, this patient's identifiable information has not been included in the service evaluation study.

## 3. Results

### 3.1. Response Times

A summary of the mean response times for CFRs and ambulances to each category of chief complaint is shown in Table 1. Eight-minute dispatch calls were those classed as immediately life-threatening, as interpreted by the call handler, for example, life-threatening haemorrhage.

Ambulance response time targets are a performance indicator for service provision in the UK [10]. These include sub-8 and sub-19 minute targets for highly urgent calls and less urgent calls, respectively. It should be noted that these are useful in some respects, such as performance monitoring; there is little consistent evidence base for these exact times [11]. CFRs and ambulance crew performance is shown in Table 2.

Therefore, CFR responses were within target in a larger proportion of attended calls compared to ambulance crew responses.

**Figure 1:**
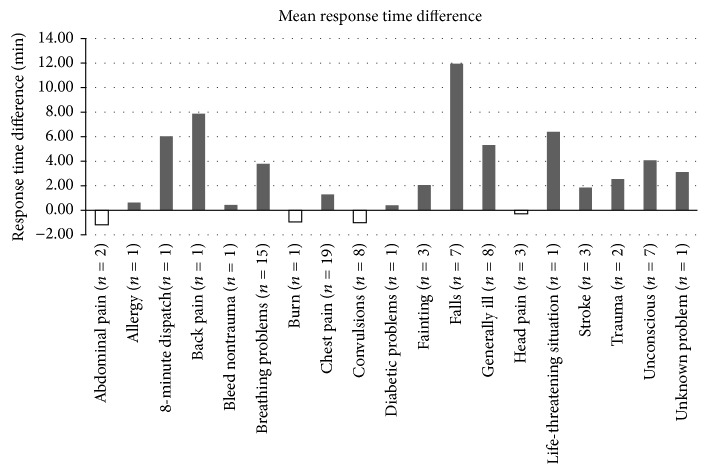
Mean response time difference, in minutes, between CFR and ambulance crews, separated by chief complaint.

Figure 1 shows the mean time difference between CFR and ambulance responses. This difference varied by chief complaint. Ambulance crews attended six calls relating to abdominal pain, burns, and head pain, on average, faster than the CFRs. However, on all other analyzed calls (allowing for Assumption (4)), the CFR response time was less.

**Figure 2:**
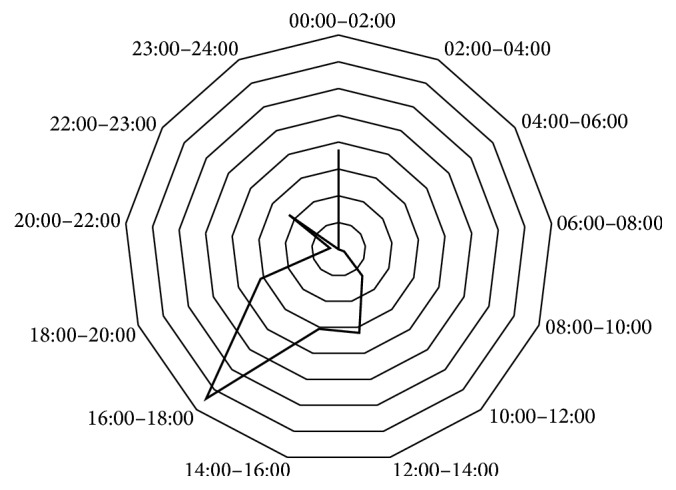
Response time differences around the clock. The largest mean response time difference (7 min, 27 s) was observed between 16:00 and 18:00. Each polygonal division represents 1 minute.

Response time differences also varied by call time. As shown in Figure 2, the largest difference (7 minutes and 27 seconds) in response times occurred in evening peak hours, between 16:00 and 18:00.

### 3.2. CFR Questionnaire

Of the twenty CFRs surveyed, twelve responded. All responders regarded the experience gained through the scheme as educationally valuable.  Table 3 shows the responses received. Additionally, the survey demonstrated that the students gained a positive sense of community involvement.

## 4. Discussion

The following assumptions were made and limitations were identified in this analysis:

1. CFR allocation time did not vary significantly from ambulance allocation time. This allowed the CFR allocation time to be used as start time for both groups. This assumption is reasonable as if there was, in practice, any variation, it is expected that ambulance crews would be allocated at the same time as CFRs, if not sooner.
2. Chief complaints included in the analysis were those recorded on taking the emergency call. It is possible that the complaints may have been different on assessment by CFRs and crews. However, it is assumed that this did not impact response times for either group.
3. For the purposes of the analysis, all calls were treated equally. Therefore, any variations in CFR and ambulance response times were not adjusted for the urgency of the call. It should be noted that triage systems are used by ambulance dispatchers which may have led to confounding errors related to call type.
4. Out of the 89 calls attended, four were excluded from the analysis. Three ambulance arrival times were excluded as the ambulances arrived prior to CFR allocation, preventing the calculation of response times. Further, in one instance, no ambulance attended the call.
5. CFR arrival time was retrospectively collected from the CFRs after the emergency call. This collection method is less accurate than the GPS based system used by the ambulance service.
6. No patient outcomes were measured.
7. No specific student educational outcomes were measured.
8. A small number of calls were analyzed, with many call categories including only one call. This reduces the power of this study.

It was noted that the response time disparity was not statistically significant for urgent calls, such as those for chest pain (1 minute and 18 seconds (95% CI −01:33 to 04:09)). It is surmised that this may be due to a number of factors:

- Ambulance crews may have been rerouted to more critical calls on receipt. Data was not available regarding this.
- Rapid response traffic maneuvers, sirens, and lights may have been used more extensively by ambulance crews in more critical calls. This may have allowed faster arrival on scene. Such tools were not available to CFRs.

Further, the availability of CFRs may have biased ambulance allocation. As the greatest decrease in mean response time related to less acute calls, it may be that CFRs were used as a low cost resource. In such cases, CFRs would provide rapid cover and an ambulance would not be dispatched immediately; rather, the ambulance would be delayed unless escalated by the CFRs. Although it is important to consider this possibility, it was not part of the scheme design and there is currently no evidence base supporting the use of CFR schemes in noncritical emergency calls. Rather, the evidence supports CFRs in emergency contexts, such as cardiac arrests, in which differences in arrival times were less significant.

It should be noted that CFR schemes were established on evidence pertaining to urgent calls, especially out of hospital cardiac arrests [12]. The analysis presented here suggests that the largest benefit, in terms of average response times, is seen for nonurgent calls. This should be considered when considering implementing such schemes: there is scope to improve service delivery for a broad range of calls. It should be determined whether CFR intervention improves patient outcomes for nonurgent calls; however, this was beyond the scope of this analysis.

The largest mean response time difference was between the afternoon peak hours of 16:00 and 18:00 (7 minutes and 27 seconds). It is proposed that this observation was due the proximity of the CFR to the scene and due to increased congestion on the roads.

Patient outcomes were not measured in this study. This limitation precludes determination of any benefit or harm for patients as result of attendance of and treatment by CFRs. It has been assumed that there was no additional risk to patient safety, with training provided to mitigate risk. Further studies must be undertaken to elucidate this.

In addition to providing reduced response times, the CFR scheme was also seen as a beneficial educational experience. Participants in the scheme gained confidence in dealing with real-life medical emergencies and stated that both the training and hands-on experience positively contributed to their acute and emergency medicine education. By integrating such schemes into medical school curricula, it is proposed that future junior doctors will be better prepared for managing the acutely unwell patient.

There are a number of limitations of this work. Firstly, no data on patient outcomes was recorded. Colleting this data in future work will allow any benefit of such a CFR scheme, beyond response times alone, to be elucidated. Secondly, the scheme covered a relatively small number of calls. Lastly, the scheme was carried out in a single geographical region. By including other regions in future work, the national and international applicability of the presented findings could be ascertained.

## 5. Conclusion

A CFR group, comprising final and penultimate year UK medical students trained to the First Person on Scene First Aid standard [13], was successfully trained and deployed in the inner-city Birmingham (UK) area. The mean CFR response time across all calls was an average of 3 minutes and 8 seconds (95% CI 01:02 to 05:14) less than ambulance crew response times, representing a statistically significant reduction in time to first on scene. The largest difference in the mean response times, when categorized by chief complaint, was between the responses to calls relating to falls (12 min).

## Figures and Tables

**Table 1:** Mean response times of CFR and ambulance crews by chief complaint, showing a statistically significant (at the 95% confidence level) mean decrease of 3 minutes and 8 seconds, comparing ambulance response times to CFR response times.

Chief complaint	*n* ^a^	Mean CFR response time/min	Mean ambulance response time/min	Difference in mean response time (ambulance – CFR)/min
Generally ill^c^	8 (10)	10:54	16:12	05:19
Falls^b^	7 (8)	09:37	21:34	11:57
Unknown problem^d^	1 (2)	05:31	08:38	03:07
Chest pain	19	08:03	09:21	01:18
Breathing problems	15	07:59	11:47	03:48
Convulsions	8	06:47	05:47	−01:00
Unconscious	7	05:34	09:39	04:05
Fainting	3	06:38	08:42	02:04
Head pain	3	10:44	10:27	−00:17
Stroke	3	10:51	12:43	01:52
Abdominal pain	2	15:29	14:19	0
Trauma	2	06:34	09:07	02:33
Allergy	1	10:33	11:11	00:38
Ambulance dispatch, 8 minutes	1	01:53	07:55	06:02
Back pain	1	11:03	18:55	07:52
Bleeding, nontraumatic	1	12:48	13:15	00:27
Burn	1	06:01	05:05	−00:56
Diabetic problems	1	03:49	04:14	00:25
Life-threatening situation	1	04:19	10:43	06:24

Overall	**85 **(89)	**08:18**	**11:25**	**03:08**
Confidence interval at 95% Confidence level		[07:11, 09:25]	[09:38, 13:12]	[01:02, 05:14]

^a^ *n* = number of calls analyzed in this category. Figures in parenthesis, (#), indicate the total number of calls recorded, as per Assumption (4) in Section 4. ^b^One ambulance arrival time was excluded as the vehicle arrived before the CFR allocation time. ^c^Two ambulance arrival times were excluded as the vehicles arrived before the CFR allocation times. ^d^One call attended only by CFRs.

Over the 89 calls, the CFR group achieved an average response time of 8 minutes and 18 seconds (95% CI 07:11 to 09:25). This compares to the ambulance crew average response time of 11 minutes and 25 seconds (95% CI 09:38 to 13:12), presenting a statistically significant 3 minutes and 8 seconds of (95% CI 01:02 to 05:14) additional delay. Further, CFRs were first on scene in 59 out of 85 calls (69%; data not shown).

**Table 2:** CFR and ambulance crew response time performance against current targets, showing the larger proportion of within-target response times for CFRs versus ambulance crews. Note that these results are indicative of response time only and do not reflect the target category of the call itself.

	Under 8 minutes	Under 19 minutes	Over 19 minutes	Total
Community First Responders (*n* = 85)	49	31	5	85
	*55%*	*35%*	*5%*	

Ambulance crews (*n* = 85)	30	48	7	85
	*35%*	*57%*	*8%*	

**Table 3:** Student responses to qualitative survey on educational value and community contribution (*n* = 12).

Question	Response	
	Yes	No
Having trained as a CFR do you feel more confident assessing and treating critically unwell patients? (this question relates solely to the training weekend)	12	0
Having volunteered as a CFR do you feel more confident assessing and treating critically unwell patients?	12	0
Do you feel the CFR work prepared you for the Acutely Unwell Patient OCSE station?	10	2
Do you think that the CFR scheme positively added to your medical education?	12	0
Did you find it rewarding giving back to the local community?	12	0

## Abbreviations

CFR: Community First Responder
UK: United Kingdom
WMAS: West Midlands Ambulance Service.
